# Application and Research Progress of High Frequency Ultrasound in the Diagnosis of Chronic Inflammatory Neuropathies

**DOI:** 10.3389/fneur.2022.860144

**Published:** 2022-06-23

**Authors:** Xishun Ma, Lizhen Du, Wenqing Yuan, Tongliang Han

**Affiliations:** ^1^Department of Ultrasound, Qingdao Municipal Hospital, Qingdao, China; ^2^Department of Primary Medical Management, Qingdao Municipal Hospital, Qingdao, China

**Keywords:** chronic inflammatory neuropathies, chronic inflammatory demyelinating polyneuropathies, multifocal motor neuropathy (MMN), Lewis-Sumner syndrome, high frequency ultrasound

## Abstract

In recent years, clinicians have gradually improved their understanding of multiple neuropathy and have done some studies about chronic inflammatory neuropathies, for example, chronic inflammatory demyelinating polyneuropathy, multifocal motor neuropathy, and Lewis-Sumne syndrome. The early diagnosis is very important for the next step treatment and long-term prognosis. At present, the disease mainly depends on clinical and neural electrophysiological examination, but imaging studies are few. In recent years, with the rapid development of high frequency ultrasound, it could clearly show the morphology of the nerve, and it has been an emerging diagnosis tool of polyneuropathies. This article mainly reviews the application and the latest research progress of high frequency ultrasound in these diseases.

## Introduction

The diagnosis of chronic inflammatory neuropathies could be challenging (1, 2). The current diagnostic criteria are mainly based on both clinical and electrophysiological parameters. Particularly in chronic inflammatory neuropathies cases with atypical clinical presentation or at early stages of the diseases, the early diagnosis rate is low, and it lacks quantitative criteria for assessing disease activity and treatment response, leading to severely affecting long-term prognostic effects of such diseases. Recent studies have shown that nerve ultrasound has a high sensitivity and acceptable specificity in a cohort of consecutive patients with a clinical suspicion of chronic inflammatory demyelinating polyneuropathy, Lewis-Sumner syndrome, and multifocal motor neuropathy, thereby improving identification of patients who may respond to treatment (3–5). Exploring the imaging characteristics of high-frequency ultrasound could help to establish combined diagnostic criteria for chronic inflammatory neuropathies, improve the accuracy of early diagnosis, and facilitate the follow-up of diseases.

## The Ultrasound Research Process of Chronic Inflammatory Neuropathies

### Chronic Inflammatory Demyelinating Polyneuropathy

#### The Ultrasound Imaging Features of Chronic Inflammatory Demyelinating Polyneuropathy

The chronic inflammatory demyelinating polyneuropathy is an immune-mediated chronic sensorimotor polyneuropathy characterized by proximal limb weakness, weakened tendon reflex, and sensory abnormalities in distal limb, but it also includes rare clinical variants such as simple sensory abnormalities, simple motor abnormalities, and limb asymmetry (6, 7). High-frequency ultrasound can probe the morphological changes in the peripheral nerves to indirectly reflect the neuropathological changes of chronic inflammatory demyelinating polyneuropathy, so it could contribute to diagnosis and treatment (8–10). Several studies confirmed that nerve enlargement occurred in over 90% of patients with chronic inflammatory demyelinating polyneuropathy whether treated or not, but it had a different degree and morphologies. Specific results showed that the cross-sectional area of new chronic inflammatory demyelinating polyneuropathy and long-term-treated chronic inflammatory demyelinating polyneuropathy increased. The ultrasound pattern sum score system: chronic inflammatory demyelinating polyneuropathy scores were higher than new chronic inflammatory demyelinating polyneuropathy. It had also shown that newly developed chronic inflammatory demyelinating polyneuropathy nerve enlargement was mostly localized, however, chronic inflammatory demyelinating polyneuropathy was generalized. The uniformity score results showed that chronic inflammatory demyelinating polyneuropathy had higher points than new chronic inflammatory demyelinating polyneuropathy (11, 12). Nerve enlargement mainly occurred in the upper limb proximal nerve segments and the arm plexus (13, 14), and ultrasound had a high diagnostic rate of evaluation of nerves in these areas (13). In addition to nerve enlargement, there were increased blood flow signals, enhanced echo or disappearance of normal nerve structures (8, 14). Most studies had found a relationship between nerve enlargement and slow motor conduction velocity (9). Padua and colleagues found that nerve enlargement was more pronounced in patients with longer disease duration and that echo at the disappearance of normal nerve tract structure was decreased (14).

In addition, some studies found that chronic inflammatory neuropathies had higher echoes compared to axonal neuropathy in the ulnar nerve of the forearm, the median nerve in the forearm and upper arms, and the arm plexus, which was statistically significant, and it could contribute to the identification of disease. Since most studies are monocentric, further multicentre studies of the ultrasound aspects of chronic inflammatory demyelinating polyneuropathy are required.

**Figure 1:**
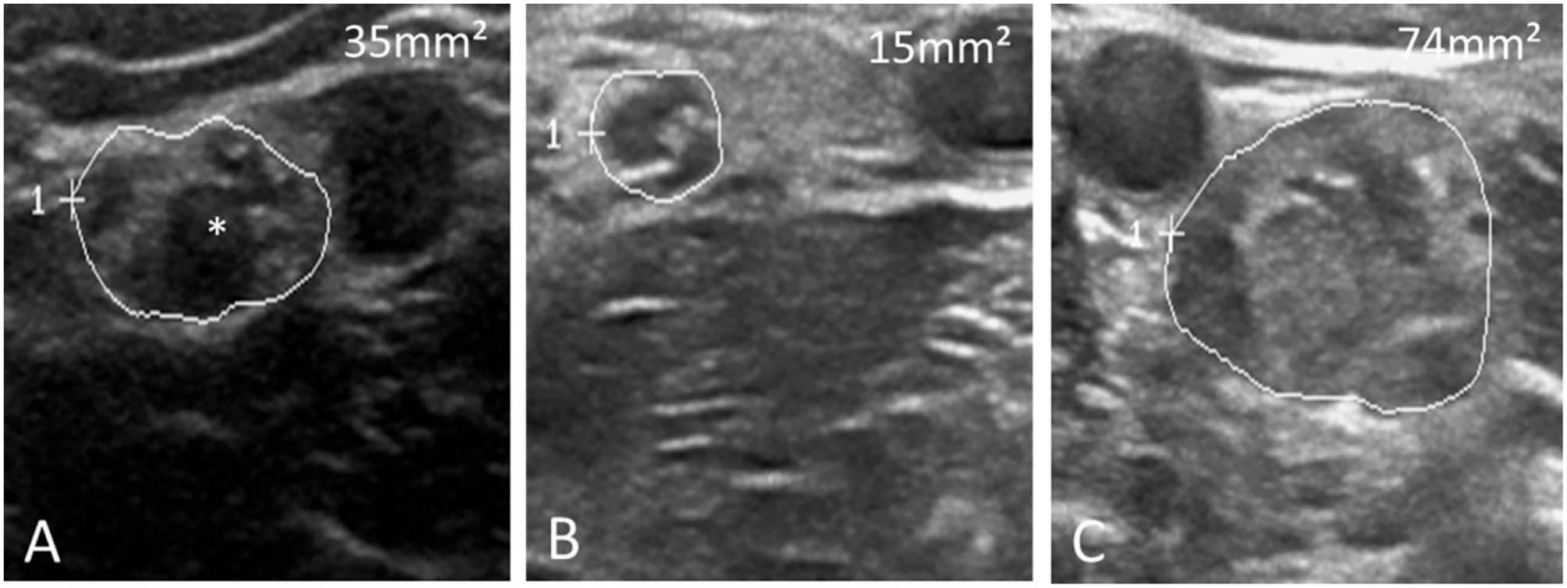
Three distinct ultrasound morphologies in chronic inflammatory demyelinating polyneuropathy. **(A)** Shows an enlarged median nerve (MN, CSA 35 mm^2^, normal values 13 mm^2^) with reduced echointensity and enlarged fascicles (*), while **(B)** Shows an only slightly enlarged nerve without fascicle enlargement (CSA 15 mm^2^). In contrast, in **(C)** the MN is tremendously enlarged (74 mm^2^) with otherwise hyperechoic intraneural echo signature. **(A)** Resembles the most common nerve morphology in chronic inflammatory demyelinating polyneuropathy.

**Figure 2:**
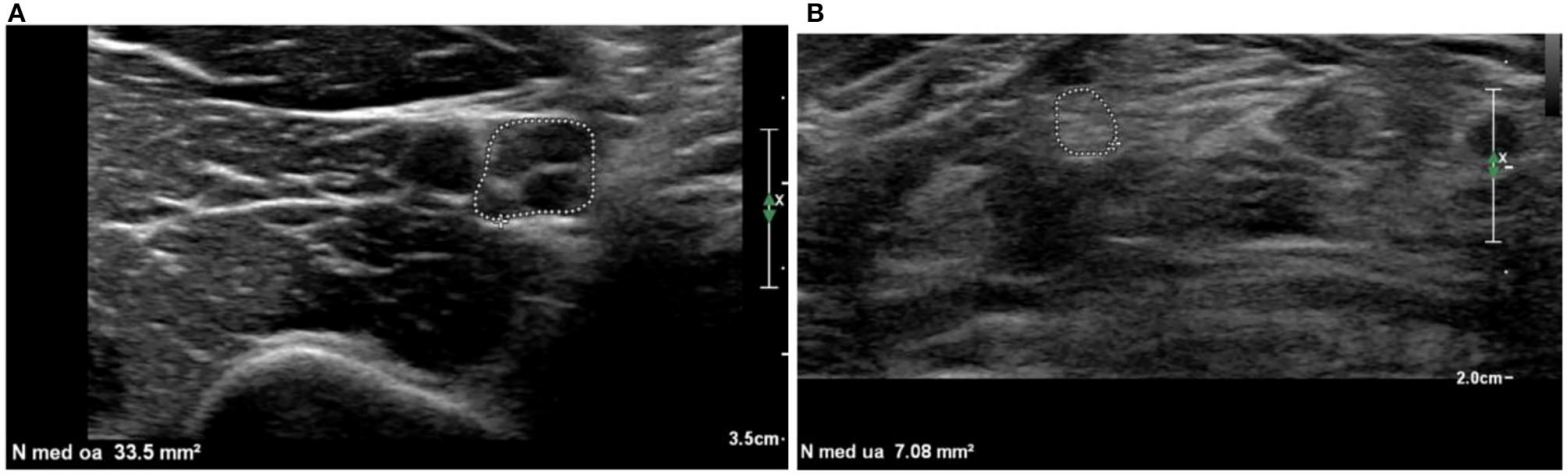
Examples of a hypoechoic **(A)** and hyperechoic **(B)** nerve. **(A)** Median nerve at the upper arm is enlarged (CSA = 33.5 mm^2^) and shows enlarged hypoechoic fascicles (fraction of black = 85%). **(B)** Median nerve at the forearm with CSA in normal range, hyperechoic (fraction of black = 9%), fascicles are difficult to distinct. Hyperechoic nerves as in **(B)** can be difficult to distinguish from the surrounding structures. Continuous examination of the whole length of the nerve and identification based on the known anatomical course is necessary to ensure proper identification of the nerve and the nerve outline.

Specific cases were shown in Figures 1, 2 (15, 16).

#### Monitoring Disease Treatment Response With HFUS

A scoring tool of standardized method or Ultrasound Pattern Sum Score can better monitor the treatment response, specifically, the changes before and after treatment can be quantified by measuring the cross-sectional area of nerves. Some studies reported that the extent of nerve enlargement attenuated or disappeared after a period of treatment (17). On the other hand, it had been observed that patients with hypoecho nerve enlargement had better treatment effect compared to those with no nerve enlargement or increased echo (13, 14).

### Multifocal Motor Neuropathy

#### The Ultrasound Imaging Features of Multifocal Motor Neuropathy

Multifocal motor neuropathy is an immune-mediated, simple motor neuropathy characterized by slow disease progression, a typical limb asymmetry muscle weakness that can be accompanied by muscular atrophy (18–21). The characteristic electrophysiological examination criterion for multifocal motor neuropathy is the conduction block at common neurojammed sites (22). Nerve ultrasound imaging features contribute to diagnosis and differential diagnosis of multifocal motor neuropathy.

Nerve enlargement of multifocal motor neuropathy, which often represents mild multifocal and asymmetry compared to chronic inflammatory demyelinating polyneuropathy, was mainly found in the arm plexus and large peripheral nerve in the extremities (23, 24).

The study showed that multifocal nerve enlargement could distinguish multifocal motor neuropathy from healthy control subjects with a sensitivity of 87–100% and a specificity of 94–100% (25–27). The ratio of CSA differences of intra-and internerve and one side and other side [two ultrasound scoring systems (28, 29)] was also increased in multifocal motor neuropathy patients (24, 30). Individual nerve bundles within the nerve may also have varying degrees of enlargement, while adjacent bundles are intact (31). Thus, asymmetric multifocal nerve enlargement may indicate multifocal motor neuropathy, but further studies remain to determine the specificity of this finding, compared to other polynerves, such as asymmetry variants of chronic inflammatory demyelinating polyneuropathy. Some studies showed that the multifocal motor neuropathy group was more pronounced in the median forearm nerve, the ulnar nerve, and the tibia nerve, compared with normal controls. Neuroelectrophysiological examination indicated that it had lower nerve conduction velocity and compound muscle action potential at the upper arm nerve. The multifocal motor neuropathy group had important relationship between CSA of the median nerve in the upper arm and the compound muscle action potential (correlation *r* = 0.851). The multifocal motor neuropathy group had a higher “difference ratio of CSA between one side and the other” in the median, ulnar, and fibula nerve levels, with no significant change in the tibial and brachial plexus levels. Compared with chronic inflammatory demyelinating polyneuropathy, multifocal motor neuropathy patients predominantly showed reduced echo of nerve enlargement areas (31). It rarely found increased echo compared to Lewis-Sumner syndrome and chronic inflammatory demyelinating polyneuropathy, which may contribute to the differential diagnosis of multifocal motor neuropathy and other major motor neuropathies.

#### Monitoring Disease Treatment Response With HFUS

Recently, it is rarely known about the treatment response and ultrasound application development of multifocal motor neuropathy. A recent study, following up 17 treated multifocal motor neuropathy patients for several months, firstly, demonstrated the degree of nerve enlargement was not proportional to clinical presentation, and secondly, the Ultrasound Pattern Sum Score score demonstrated a significant correlation between changes in motor nerve dysfunction and changes in ultrasound imaging regardless of the disease development (32).

### Lewis-Sumner Syndrome

#### The Ultrasound Imaging Features of Lewis-Sumner Syndrome

Lewis-Sumner syndrome belongs to a variant of chronic inflammatory demyelinating polyneuropathy, an autoimmune disease, mainly showing limb asymmetric sensory, motor peripheral neuropathy. The most commonly involved nerves are the median nerve and the ulnar nerve. The electrophysiological examination has the motor nerve block, and the pathological characteristics are mainly mild demyelinating lesions.

No studies analyzed lewis-sumner syndrome as a single entity, but many studies described its ultrasound performance second only to chronic inflammatory demyelinating polyneuropathy and multifocal motor neuropathy (33). Overall, researchers agreed that most Lewis-Sumner syndrome patients had nerve enlargement. In many cases, neuromorphological changes were similar to chronic inflammatory demyelinating polyneuropathy, with nerve enlargement ranging from mild to widespread. However, recent studies showed that Lewis-Sumner syndrome nerve enlargement was mainly regional and limited to a single major nerve tract, rarely affecting asymmetric distributed nerve in the arm and roots (34).

Interestingly, the echo intensity of enlarged nerves usually decreased, but sometimes also increased, with as described by chronic inflammatory demyelinating polyneuropathy, and the cranial nerves were more susceptible in Lewis-Sumner syndrome than in chronic inflammatory demyelinating polyneuropathy, such as vagus enlargement (35, 36). In summary, the Lewis-Sumner syndrome ultrasound morphology behaved similar to an overlapping chronic inflammatory demyelinating polyneuropathy and multifocal motor neuropathy. This may explain the intermediate treatment response and Lewis-Sumner syndrome clinical presentation, sometimes like typical chronic inflammatory demyelinating polyneuropathy, sometimes like multifocal motor neuropathy. However, Lewis-Sumner syndrome generally reflected nerve enlargement of the sensory nerve, which can be distinguished from multifocal motor neuropathy (25).

**Figure 3:**
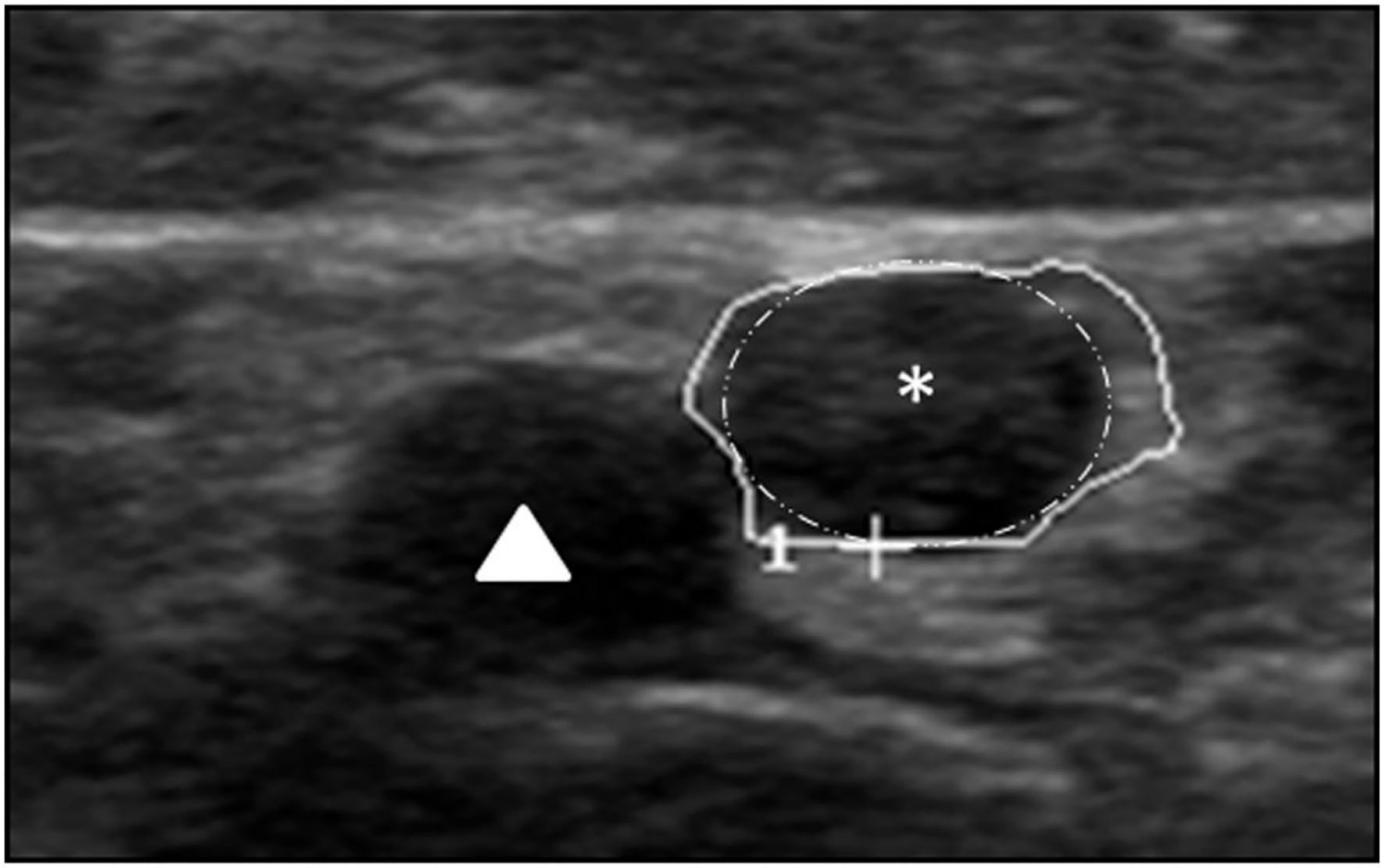
This shows a regionally predominant fascicle enlargement (white surrounding with star) with 9 mm^2^ next to normal fascicles in the median nerve (14 mm^2^) in a patient with Lewis-Sumner syndrome, accompanied by the brachial artery (triangle). The enlarged fascicle almost covers the whole CSA.

Specific cases were shown in Figure 3 (32).

#### Monitoring Disease Treatment Response With HFUS

With regard to ultrasound changes during treatment, most investigators found recoverance of enlarged nerves in response to treatment (17, 37). It had also been reported that nerve enlargement continued with despite improved clinical symptoms (36). However, prospective studies of disease and ultrasound monitoring remain unknown.

## The Clinical Ultrasound Scoring System

### Bochum Scoring

Measuring: the ulnar nerve in the Guyon canal; the ulnar nerve in the upper arm; the radial nerve in the spiral groove; the sural nerve in the calf

Recording: every site scored one point, then added together

Results: identification of chronic inflammatory demyelinating polyneuropathy from multifocal motor neuropathy and Lewis-Sumner syndrome (CSA cutoff value≥ 2). Sensitivity was 80%, and specificity was 87.5% (38).

### Ultrasound Pattern Sum Score Scoring

Measuring: median nerve of upper arm, elbow and forearm; ulnar nerve of upper arm and forearm; tibial nerve of popliteal fossa and ankle; peroneal nerve of popliteal fossa; vagus; C5 nerve root; C6 nerve root; sural nerve of calf

Recording:

UPS-A: measuring median nerve, ulnar nerve, tibial nerve, if nerve enlargement <50%, it scored one point. If nerve enlargement >50%, it scored two points.

UPS-B: measuring vagus, C5 and C6 nerve root, every site of nerve enlargement scores one point.

UPS-C: measuring sural nerve, every site of nerve enlargement scores one point.

UPSS: the scores sum of UPS-A, UPS-B and UPS-C

Results: UPSS ≥ 10 or UPS-A ≥ 7 indicated chronic inflammatory demyelinating polyneuropathy. 3 < UPSS < 10 and UPS-B ≤ 1 indicated vascular neuropathy. UPSS ≤ 3 indicated axonal neuropathy.

Specific cases were shown Table 1 (13).

**Table 1:** The ultrasonic pattern sum score UPSS.

**Measurement**	**Boundary**	**Normal**	**Points**
	**value in mm^**2**^**	** <50%**	
		**>50%**	
**Peripheral nerves**			
Median nerve	10.0	<10	0
Upper arm		≥10 ≤ 15	1
		>15	2
Median nerve	11.5	<11.5	0
Elbow		≥11.5 ≤ 17.25	1
		>17.25	2
Median nerve	10.0	<10	0
Forearm		≥10 ≤ 15	1
		>15	2
Ulnar nerve	9.5	<9.5	0
Upper arm		≥9.5 ≤ 14.25	1
		>14.25	2
Ulnar nerve	8.5	<8.5	0
Forearm		≥8.5 ≤ 12.75	1
		>12.75	2
Tibial nerve	29.5	<29.5	0
Popliteal		≥29.5 ≤ 44.25	1
		>44.25	2
Tibial nerve	10.5	<10.5	0
Ankle		≥10.5 ≤ 16.25	1
		>16.25	2
Fibular nerve	11.5	≤11.5	0
Popliteal		≥11.5 ≤ 17.25	1
		>17.25	2
**Cervical roots and vagus**			
Vagal nerve	3.0	≤3.0	0
Carotid sheath		>3.0	1
C5 longitudinal	2.9 (mm)	≤2.9	0
Transversal process		>2.9	1
C6 longitudinal	3.9 (mm)	≤3.9	0
Transversal process		>3.9	1
**Sural nerve**			
Sural nerve	3.0	≤3.0	0
Calf		>3.0	1
Ultrasound pattern sum score			

*UPS-A. Nerve score: each nerve swelling is assessed with 1 point, each swelling > 50% of the boundary value is assessed with 2 points. UPS-B. Root score: each root swelling or vagal nerve swelling is assessed with 1 point. UPS-C. Sural score: sural nerve swelling is assessed with 1 point. UPSS, ultrasound pattern sum score*.

### Hhomogeneity Scoring

Measuring: median nerve of forearm, elbow and upper arm; ulnar nerve of forearm and upper arm; tibial nerve of popliteal fossa and ankle

Recording:

0 point: no or regional nerve enlargement; one point:heterogenicity nerve enlargement (enlarged CSA < 50% or > 50%); two points: mild homogeneity nerve enlargement(enlarged CSA < 50%); three points: obvious homogeneity nerve enlargement(enlarged CSA > 50%).

Results: In chronic inflammatory demyelinating polyneuropathy the enlargement was regional, homogeneous, or inhomogeneous with equal contribution. In multifocal motor neuropathy rebional enlargement next to normal segments predominated (25).

### Intra-and Internerve CSA Variabiity and Difference Ratio Between One Side and the Other Side

Measuring: any of the two nerves

Recording: Intranerve variability: the ratio of the maximum and the minimum CSA. Internerve variability: the ratio of the maximum and minimum of intranerve variability. The difference ratio between one side and other side: the ratio of maximum and minimum nerve variability.

Results: The ratio of intra-and internerve of multifocal motor neuropathy were higher than chronic inflammatory demyelinating polyneuropathy. Compared with multifocal motor neuropathy, chronic inflammatory demyelinating polyneuropathy had a higher ratio of intra-and internerve, but its ratio of one side and other side were lower.

Specific cases were shown Tables 2, 3 (29).

**Table 2:** Ultrasound findings in the median nerve at standard sites.

		**CSA, wrist**		**CSA, forearm**		**CSA, arm**		**CSA, axilla**	
**Patient number**	**Neuropathy**	**Right**	**Left**	**Right**	**left**	**Right**	**Left**	**Right**	**Left**
1	CIDP	**16**	**12**	**10**	6	**16**	**13**	**16**	**14**
2	CIDP	**15**	**14**	**22**	**21**	**42**	**29**	**32**	**19**
3	MMN	11	**15**	**10**	**17**	**19**	**28**	**23**	**22**
4	MMN	7	8	5	5	5	10	8	8
5	Anti-MAG	9	7	6	**9**	**12**	**13**	15	10
6	Anti-MAG	**13**	10	8	**9**	7	10	8	10

*CSA measured in mm^2^. Abnormal values shown in bold*.

**Table 3:** Ultrasound findings in the ulnar nerve at standard sites.

		**CSA, wrist**		**CSA, forearm**		**CSA, elbow**		**CSA, arm**		**CSA, axilla**	
**Patient number**	**Neuropathy**	**Right**	**Left**	**Right**	**left**	**Right**	**Left**	**Right**	**Left**	**Right**	**Left**
1	CIDP	6	5	7	7	**16**	**8**	**11**	**12**	**15**	**12**
2	CIDP	**14**	**10**	**18**	**21**	**13**	**17**	**17**	**24**	**21**	**18**
3	MMN	7	4	6	**17**	**14**	**19**	**15**	4	**12**	6
4	MMN	4	6	4	5	**14**	5	**60**	8	6	3
5	Anti-MAG	4	5	6	**9**	**10**	7	**9**	**8**	8	8
6	Anti-MAG	4	4	7	**10**	7	5	7	8	6	9

*CSA measured in mm^2^. Abnormal values are shown in bold*.

## Summary

As clinicians' awareness of chronic inflammatory neuropathy gradually increases, and the continuous development of high-frequency ultrasound, there are increasing studies about the clinical application of nerve ultrasound. The researchers explored the ultrasound characteristics of the disease, and it had a high specificity and sensitivity. The chronic inflammatory neuropathies mainly show nerve enlargement, increased blood flow signals, enhanced echo, or disappearance of normal nerve structures, morever, there are some few differences of them. However, the current study was small-sample studies, there were still limitations, and large-sample multicenter studies are needed in the future. Moreover, researchers should combine with the new ultrasound technology, such as elastography, ultrasound imaging, microvascular imaging, to continue to explore the ultrasound manifestations of such diseases, optimize the neural ultrasound scoring system, quantifying the diagnostic criteria for nerve ultrasound as well as the post-treatment evaluation system to better serve the clinical practice and meet the requirements of precision medicine.

## Author Contributions

All authors listed have made a substantial, direct, and intellectual contribution to the work and approved it for publication.

## Funding

This work was supported by grants from Qingdao Medical Research Guidance Plan of 2020 (No. WJZD018).

## Conflict of Interest

The authors declare that the research was conducted in the absence of any commercial or financial relationships that could be construed as a potential conflict of interest.

## Publisher's Note

All claims expressed in this article are solely those of the authors and do not necessarily represent those of their affiliated organizations, or those of the publisher, the editors and the reviewers. Any product that may be evaluated in this article, or claim that may be made by its manufacturer, is not guaranteed or endorsed by the publisher.
